# Dr. Samuel R. Cochrane

**Published:** 1902-07

**Authors:** 


Dr. Samuel R. Cochrane, of Albion, U. of B. '67, died in his office of apoplexy May 24, 1902, aged 63 years. Dr. Cochrane was one of the most prominent physicians of his region and has been a successful practitioner for more than 25 years. Besides a widow and one sister he leaves a brother, Dr. Royal E. Cochrane, of Penfield.

